# NIM: A Node Influence Based Method for Cancer Classification

**DOI:** 10.1155/2014/826373

**Published:** 2014-08-11

**Authors:** Yiwen Wang, Min Yao, Jianhua Yang

**Affiliations:** College of Computer Science and Technology, Zhejiang University, Hangzhou 310027, China

## Abstract

The classification of different cancer types owns great significance in the medical field. However, the great majority of existing cancer classification methods are clinical-based and have relatively weak diagnostic ability. With the rapid development of gene expression technology, it is able to classify different kinds of cancers using DNA microarray. Our main idea is to confront the problem of cancer classification using gene expression data from a graph-based view. Based on a new node influence model we proposed, this paper presents a novel high accuracy method for cancer classification, which is composed of four parts: the first is to calculate the similarity matrix of all samples, the second is to compute the node influence of training samples, the third is to obtain the similarity between every test sample and each class using weighted sum of node influence and similarity matrix, and the last is to classify each test sample based on its similarity between every class. The data sets used in our experiments are breast cancer, central nervous system, colon tumor, prostate cancer, acute lymphoblastic leukemia, and lung cancer. experimental results showed that our node influence based method (NIM) is more efficient and robust than the support vector machine, *K*-nearest neighbor, C4.5, naive Bayes, and CART.

## 1. Introduction

Cancer research is one of the major research areas in the medical field. In cancer, cells divide and grow uncontrollably, forming malignant tumors and invading adjacent parts of the body. The cancer may also spread to more distant parts of the body through the lymphatic system or bloodstream. Many things are deemed to increase the risk of cancer, including tobacco use, dietary factors, certain infections, exposure to radiation, lack of physical activity, obesity, and environmental pollutants. The famous Apple founder Steve Jobs also died of pancreatic cancer. Any method which benefits cancer treatment should receive sufficient attention.

The biggest challenge facing cancer treatment process is a means of developing individualized treatment programs for specific tumor types. Traditional diagnosis of cancer depends on the type of tissue-derived tumor cells, cell morphology, and protein markers, and biological behavior does not adequately reflect the real situation of the tumor; it is sometimes difficult to make a correct diagnosis of forecasts.

In order to gain a better insight into the problem of cancer classification, systematic approaches based on global gene expression analysis have been proposed [1, 2]. The expression level of genes is deemed to contain the keys to addressing fundamental problems relating to the prevention and cure of diseases, biological evolutionary mechanisms, and drug discovery. The recent advent of microarray technology has upheld the simultaneous monitoring of thousands of genes, which motivated the development in cancer classification using gene expression data [3]. From the data mining perspective, measuring the gene sequence to predict tumor is actually a classification problem. Due to the characteristics of gene expression data, there are three challenges for cancer classification. 

(*1) High Dimension*. Each species genome is composed of a nucleotide sequence encoding a protein and nonprotein coding; the former is the traditional sense of the gene, which is a potential gene. Usually, the number of genes is the total of both. The number of genes in the human genome is approximately 30000. The dimension of the data is so high, brought great difficulties to the analysis of the experimental results. For example, used in our experiments the maximum dimension of a data set is up to 24481 in breast cancer [4].

(*2) Small Sample Size*. Since the acquisition of gene expression experiments in extreme cost data, publicly available data size is very small. Most tumor gene expression data sample numbers of only tens or hundreds. But the traditional classification methods often require a large set of test samples to obtain the high classification accuracy. This is a huge challenge for classification algorithm. For instance, used in our experiments later cancer data central nervous system [5] has only 60 samples, but with 7129 dimensions.

(*3) Nonbalanced Distribution.* Usually, the traditional classification methods can achieve outstanding results when using balanced distribution data. However, gene microarray data for cancer classification are nonbalanced distribution. For example, in lung cancer [6] used in our experiments later, the number of samples of the MPM class is 31 and the number of samples of ADCA class is 150, which is nearly 5 times that of the former.

## 2. Node Influence Model

Let *m* indicate the number of genes measured. Every cancer sample can be viewed as a point in *m*-dimensional space. And the set of cancer samples can be viewed as a graph (or network) in *m*-dimensional space. Our idea is to confront the problem of cancer classification from graph-based view. In graph theory, a graph (or network) is usually presented by an adjacency matrix. If a graph has *N* vertices, we may associate it with an *N* × *N* matrix *A*. The adjacency matrix *A* is defined by

(1) $$\begin{array}{c} A\left(v_{i},v_{j}\right)=\{\begin{array}{cc} 1, & v_{i}\text{ and }v_{j}\text{ connected,}\\ 0, & v_{i}\text{ and }v_{j}\text{ not connected.} \end{array} \end{array}$$

### 2.1. Centrality Measures for Node Influences

The centrality of nodes, or the identification of the importance of nodes, is a key issue in network analysis. Degree is the simplest of the node centrality measures by using the local structure around nodes only. In an undirected network, the degree is equal to the number of edges a node has. In a directed network, a node may have a different number of outgoing and incoming edges, and therefore, degree is split into out-degree and in-degree, respectively. The degree centrality of a vertex *v*_*i*_, for a given graph *G* = (*V*, *E*) with |*V*| = *N* vertices and |*E*| = *M* edges, is defined as

(2) $$\begin{array}{c} \text{Degree}\left(v_{i}\right)=\overset{N}{\underset{j=1}{\sum }}\delta_{i}^{j},\\ \delta_{i}^{j}=\{\begin{array}{cc} 1, & v_{i}\text{ and }v_{j}\text{ connected,}\\ 0, & v_{i}\text{ and }v_{j}\text{ not connected.} \end{array} \end{array}$$

Closeness is defined as the inverse of farness, which in turn, is the sum of distances to all other nodes [7]. The intent behind this measure is to identify the nodes which could reach others quickly. The closeness centrality of a vertex *v*_*i*_, for a given graph *G* = (*V*, *E*) with |*V*| = *N* vertices and |*E*| = *M* edges, is defined as

(3) $$\begin{array}{c} \text{Closeness}\left(v_{i}\right)=\frac{1}{\sum_{i\neq j,v_{j}\in E}d_{ij}}, \end{array}$$

where *d*_*ij*_ is the distance of shortest path from node *v*_*i*_ to node *v*_*j*_.

Another famous node centrality is betweenness [7], a measure of how many shortest paths cross through this node, which is believed to determine who has more interpersonal influence on others. High betweenness individuals often do not have the shortest average path to everyone else, but they have the greatest number of shortest paths that necessarily have to go through them. Betweenness centrality of a vertex *v*_*i*_, for a given graph *G* = (*V*, *E*) with |*V*| = *N* vertices and |*E*| = *M* edges, is defined as

(4) $$\begin{array}{c} \text{Betweenness}\left(v_{i}\right)=\underset{v_{s}\neq v_{i}\neq v_{s}\in V}{\sum }\frac{\sigma_{st}\left(v_{i}\right)}{\sigma_{st}}, \end{array}$$

where *σ*_*st*_ is total number of shortest paths from node *v*_*s*_ to node *v*_*t*_ and *σ*_*st*_(*v*_*i*_) is the number of those paths that pass through node *v*_*i*_.

**Figure 1:**
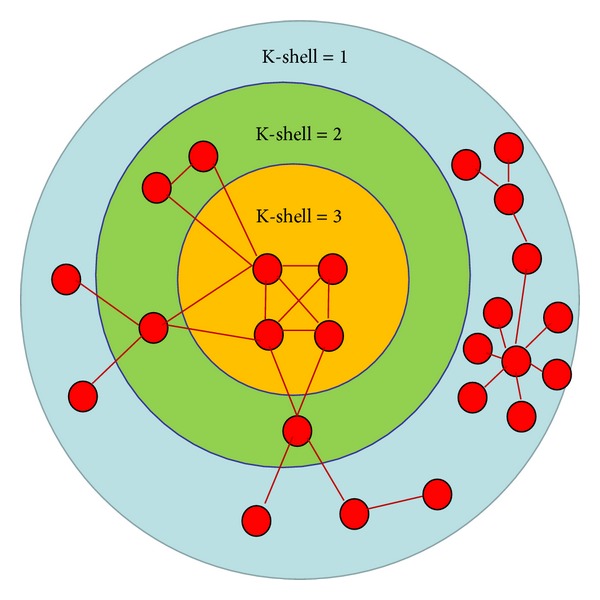
A schematic representation of the K-shell.

K-shell [8] is a relatively recent and robust centrality. Nodes are assigned to K shells according to their remaining degree, which is obtained by successive pruning of nodes with degree smaller than the K-shell value of the current layer.  Figure 1 is a schematic representation of the K-shell. The outermost circle of Figure 1 is the nodes with K-shell = 1; delete theses nodes and then consider the remaining nodes of degree 2. Then we obtain the second layer nodes with K-shell = 2. Delete the nodes of K-shell = 2; we finally obtain the innermost nodes with K-shell = 3.

### 2.2. Node Influence Centrality

As can be seen from the four above node centralities in complex networks, degree is the most intuitive and simple, but only considering local information. Both betweenness and closeness use shortest paths between every pair of nodes in the network as primary factor. K-shell approach is based on the node degree, but it is from a global perspective.

In our opinion, the evaluation of node centrality can start from the influence of a node on another node. Now consider the influence of node *v*_*i*_ on *v*_*j*_. If *v*_*i*_ can influence *v*_*j*_, that means that there are some paths which connected the two nodes from the topological view. So the number of paths connecting node *v*_*i*_ and node *v*_*j*_ is able to reflect the influence. From a global perspective, the number of connected paths between all nodes and node *v*_*j*_ must be taken into account. Therefore, we define the influence of one node on another node with length *k* as follows:

(5) $$\begin{array}{c} {\text{Influence}}_{i\to j}^{k}=\frac{\sigma_{j}^{k}\left(v_{i}\right)}{\sigma_{j}^{k}}, \end{array}$$

when *σ*_*j*_^*k*^ represents the number of connected paths between all nodes and node *v*_*j*_ with length *k* and *σ*_*j*_^*k*^(*v*_*i*_) represents the number of connected paths between node *v*_*i*_ and node *v*_*j*_. We found that, in an undirected network, when *k* tends to infinity, Influence_*i*→*j*_^*k*^ will fluctuate at the beginning and then stabilize; that is, it will converge to a certain value.

**Theorem 1 .**

When *k* tends to infinity,  *Influence*_*i*→*j*_^*k*^ will converge to a certain value in an undirected network.

**Proof**

To facilitate the proof, we introduce one good nature of adjacency matrix. That is, the *k*th power of the adjacency matrix elements represents the corresponding number of connected paths between two nodes with length *k*. Consider

(6) $$\begin{array}{c} \sigma_{j}^{k}\left(v_{i}\right)=A^{k}\left(v_{i},v_{j}\right),\\ \sigma_{j}^{k}=\overset{N}{\underset{m=1}{\sum }}A^{k}\left(v_{m},v_{j}\right). \end{array}$$

So (2) can change to

(7) $$\begin{array}{c} {\text{Influence}}_{i\to j}^{k}=\frac{A^{k}\left(v_{i},v_{j}\right)}{\sum_{m=1}^{N}A^{k}\left(v_{m},v_{j}\right)}. \end{array}$$

As we consider the undirected network, *A* is a real symmetric matrix, which can be diagonalized. That is, *A* = *P* · *D* · *P*′, *P*′ is the transpose of *P*, and *P*′ = *P* ^−1^; *P* ^−1^ is inverse of *P*. *D* is a diagonal matrix, whose elements are the eigenvalues of the matrix *A*; *P* is the corresponding eigenvector. So,

(8) $$\begin{array}{c} A^{k}={\left({PDP}^{\prime }\right)}^{k}={\left({PDP}^{-1}\right)}^{k}={PD}^{k}P^{\prime },\\ D^{k}=\left(\begin{array}{cccc} d_{1}^{k} & & & \\ & d_{2}^{k} & & \\ & & \cdots & \\ & & & d_{N}^{k} \end{array}\right), \end{array}$$

*A* ^*k*^(*v*_*i*_, *v*_*j*_) = *d*_1_^*k*^ *P*_*v*_*i*_1_ *P*_*v*_*j*_1_ + *d*_2_^*k*^ *P*_*v*_*i*_2_ *P*_*v*_*j*_2_ + ⋯+*d*_*N*_^*k*^ *P*_*v*_*i*_*N*_ *P*_*v*_*j*_*N*_ = ∑_*n*=1_^*N*^ *d*_*n*_^*k*^ *P*_*v*_*i*_*n*_ *P*_*v*_*j*_*n*_, so we get Influence_*i*→*j*_^*k*^ = *A* ^*k*^(*v*_*i*_, *v*_*j*_)/∑_*m*=1_^*N*^ *A* ^*k*^(*v*_*m*_, *v*_*j*_) = ∑_*n*=1_^*N*^ *d*_*n*_^*k*^ *P*_*v*_*i*_*n*_ *P*_*v*_*j*_*n*_/∑_*m*=1_^*N*^∑_*n*=1_^*N*^ *d*_*n*_^*k*^ *P*_*mn*_ *P*_*v*_*j*_*n*_; let *d*_max⁡_ be the largest absolute value of eigenvalues of matrix *A*; then

(9) Influencei→jk=∑n=1N(dnk/dmax⁡k)PvinPvjn∑m=1N∑n=1N(dnk/dmax⁡k)PmnPvjn=Pvimax⁡Pvjmax⁡∑m=1NPmmax⁡Pvjmax⁡=Pvimax⁡∑m=1NPmmax⁡(k⟶∞)

if *d*_max⁡_ is *t*-repeated characteristic roots, and *P*_max⁡_ is the corresponding eigenvector associated with *t*-repeated roots. Consider

(10) $$\begin{array}{c} {\text{Influence}}_{i\to j}^{k}=\frac{\sum_{w=1}^{t}P_{v_{i}\max_{w}}}{\sum_{m=1}^{N}\sum_{w=1}^{t}P_{m\;\max_{w}}},\;\left(k⟶\infty \right). \end{array}$$

There is a special case that the largest absolute eigenvalues of matrix *A* are two opposite numbers. But this only happens in bipartite graph [9] and the cancer samples network is not a bipartite graph.

**Theorem 2 .**

When *k* tends to infinity, *Influence*_*i*→*j*_^*k*^ will converge to a certain value independent of the *j* in an undirected network.

**Proof**

From the proof of Theorem 1, we see (9) and (10), so Influence_*i*→*j*_^*k*^ will converge to a certain value independent of the *j*.

From Theorem 2, we know that the influence of node *v*_*i*_ on every other node in network with length *k* is the same when *k* tends to infinity. This reflects the impact of a single node *v*_*i*_ on the whole network. So we define the node influence centrality as

(11) Node Influence(vi)=Ak(vi,vj)∑m=1NAk(vm,vj), (1≤i,j≤N,k⟶∞).

### 2.3. Example for Node Influence

**Figure 2:**
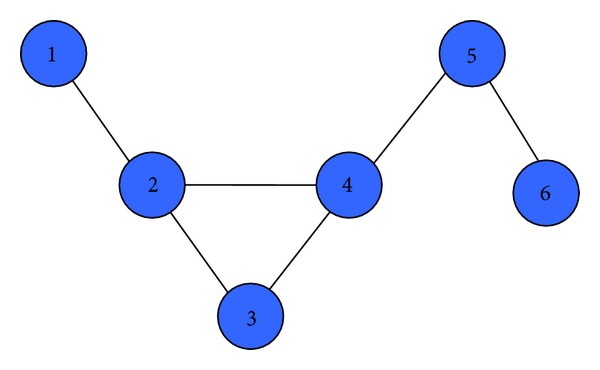
Node influence centrality example network.

For example, the network shown in Figure 2 is represented by the adjacency matrix as follows:

(12) $$\begin{array}{c} A=\left[\begin{array}{cccccc} 0 & 1 & 0 & 0 & 0 & 0\\ 1 & 0 & 1 & 1 & 0 & 0\\ 0 & 1 & 0 & 1 & 0 & 0\\ 0 & 1 & 1 & 0 & 1 & 0\\ 0 & 0 & 0 & 1 & 0 & 1\\ 0 & 0 & 0 & 0 & 1 & 0 \end{array}\right]. \end{array}$$

**Figure 3:**
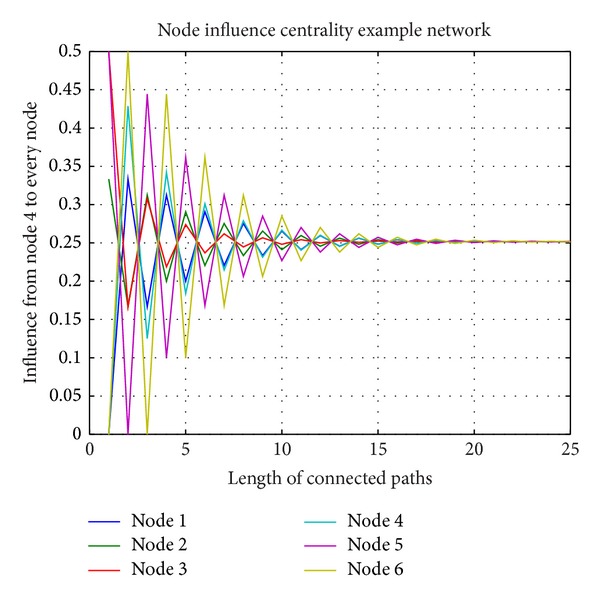
Node 4 to each node's influence.

According to (5), we calculate node 4 to each node's influence. Curves are shown in Figure 3. Curves with different colors represent the influence from node 4 every different node. From Figure 3, we can see that the influence from node 4 on each node flickers at the beginning and finally converges to about 0.25 (accurate 0.2517). This result is consistent with Theorem 2.

**Figure 4:**
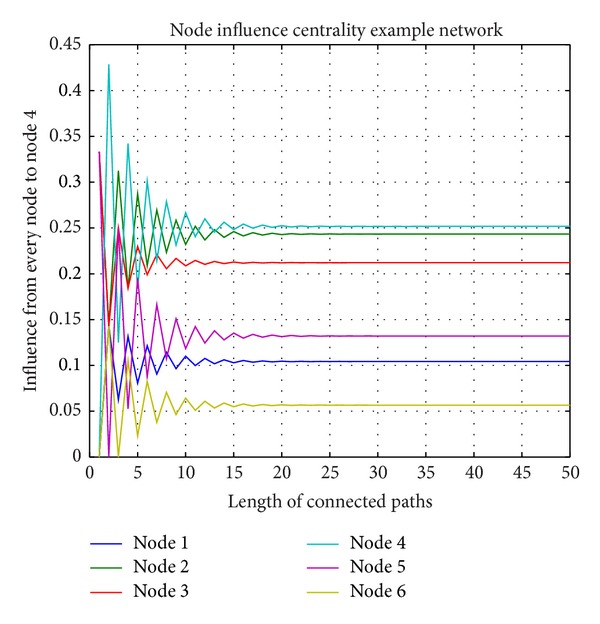
Each node to node 4 influence.

We also calculated the influence of each node on node 4, curves as shown in Figure 4. Curves with different colors represent the influence from each node on node 4. From Figure 4, we can see that the influence from each node on node 4 flickering at the beginning finally converges to different value. It is obvious that the result is consistent with Theorem 1.

## 3. Methods

### 3.1. Similarity Matrix

Let *m* indicate the number of genes measured. Every cancer sample can be viewed as a point in *m*-dimensional space. Let *N* indicate the number of samples. The according cancer samples network can be described by an *N* × *N* adjacency matrix. Edges between two nodes represent similarity between two cancer samples. For example, there are two cancer samples *X* and *Y*, *X* = (*x*_1_, *x*_2_, *x*_3_,…, *x*_*m*_), *Y* = (*y*_1_, *y*_2_, *y*_3_,…, *y*_*m*_). The weight of edge between *X* and *Y* is defined as follows:

(13) $$\begin{array}{c} \text{Similarity}\left(X,Y\right)=\exp\left(-\frac{\text{Dist}\left(X,Y\right)}{2\delta^{2}}\right), \end{array}$$

where the Dist(*X*, *Y*) is the distance metric function for two cancer samples. There are various distance metric functions. And Euclidean distance is a commonly used measure of distance when the prior knowledge is absent. Consider

(14) $$\begin{array}{c} {\text{Dist}}_{\text{Eu}}\left(X,Y\right)=\sqrt{\overset{m}{\underset{i=1}{\sum }}{\left(x_{i}-y_{i}\right)}^{2}}. \end{array}$$

After using Euclidean distance, (13) becomes

(15) $$\begin{array}{c} \text{Similarity}\left(X,Y\right)=\exp\left(-\frac{\sqrt{\sum_{i=1}^{m}{\left(x_{i}-y_{i}\right)}^{2}}}{2\delta^{2}}\right). \end{array}$$

**Figure 5:**
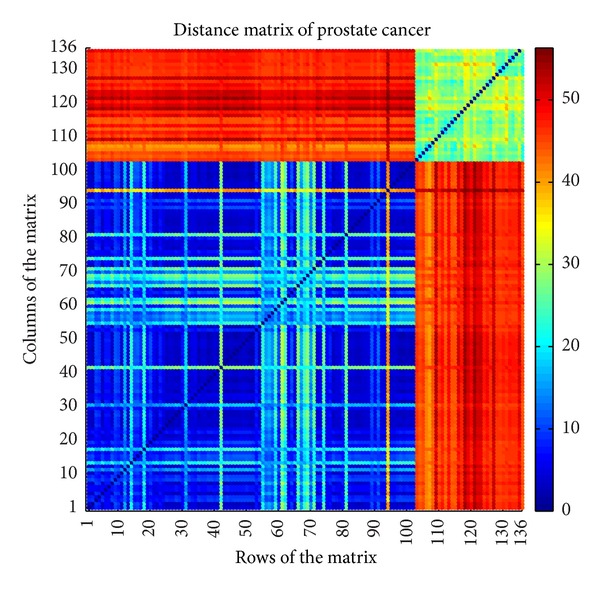
Distance matrix of prostate cancer.

**Figure 6:**
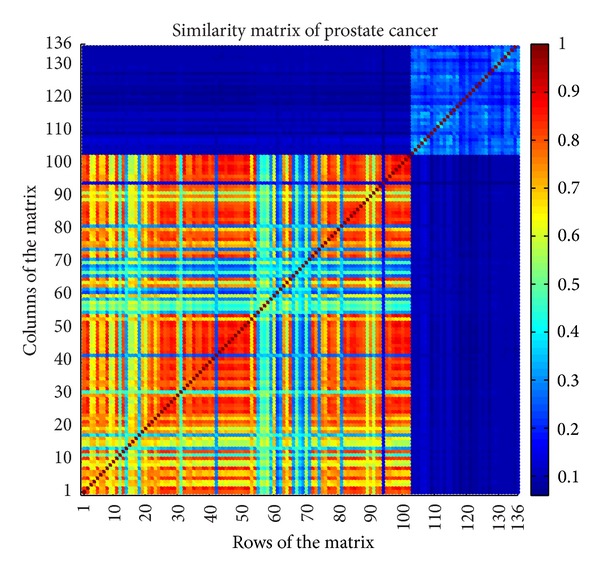
Similarity matrix of prostate cancer.

For example, the distance matrix of prostate cancer [10] described in Table 1 is shown in Figure 5. The according similarity matrix with *δ* = 3.16 is shown in Figure 6. Since there are 136 samples in prostate cancer dataset, the according distance matrix and similarity matrix are both 136 × 136.

### 3.2. Node Influence Based Method 1 (NIM1)

Node influence centrality plays a significant role in our graph-based method for cancer classification. Let *X*_train_ represent the training set, and let *X*_test_ represent the test set. All samples are divided into *n* classes, namely, *C*_1_, *C*_2_,…, *C*_*n*_. Every sample has *m* dimensions, namely, *a*1, *a*2,…, *am*. There are seven main steps in node influence based method 1 (NIM1) for cancer classification.

*Step 1*.  Data preprocessing, mainly normalization, the training set, and testing set are mapped to [0,1] range in each dimension. Only in this way can we make meaningful comparisons in later steps. Consider

(16) $$\begin{array}{c} x\cdot aj=\frac{\max\left(aj\right)-x\cdot aj}{x\cdot aj-\min\left(aj\right)},\;\left(x\in \left\{X_{\text{train}},X_{\text{test}}\right\},1\leq j\leq m\right). \end{array}$$

*Step 2*.  Select the appropriate distance metric function based on the actual problem background. If there is no prior knowledge, we recommend using the Euclidean distance. Consider

(17) Dist(x1,x2)=∑j=1m(x1·aj−x2·aj)2(x1,x2∈{Xtrain,Xtest}).

*Step 3*.  Set the only parameter *δ*; calculate the similarity between every two samples to construct the similarity matrix. Consider

(18) Similarity(x1,x2)=exp⁡(−Dist(x1,x2)2δ2)(x1,x2∈{Xtrain,Xtest}).

*Step 4*.  The training set and test set are treated as a non-negative weighted undirected network. That is, each sample in the training set or test set is treated as a node in a graph. The similarity obtained in Step 3 for every two samples is treated as the weight of the edge connecting the two corresponding nodes. Then we obtain the adjacency matrix for the whole cancer samples. Consider

(19) $$\begin{array}{c} A\left(x1,x2\right)=\text{Simlarity}\left(x1,x2\right)\;\left(x1,x2\in \left\{X_{\text{train}},X_{\text{test}}\right\}\right). \end{array}$$

*Step 5*.  Calculate the node influence centrality of each training sample node, and treat it as the weight. Consider

(20) Node Influence(xtrain)=Ak(xtrain,xa)∑x∈{Xtrain,Xtest}Ak(x,xa)(xtrain∈Xtrain,k⟶∞).

*x*_*a*_ is an arbitrary element in set {*X*_train_, *X*_test_}. Consider

(21) $$\begin{array}{c} \text{weight}\left(x_{\text{train}}\right)=\text{Node Influence}\left(x_{\text{train}}\right). \end{array}$$

*Step 6*.  Calculate the similarity between every test sample and each class. Consider

(22) Similarity Class(xtest,Ci)=∑Similarity(xtest,xtrain)·weight(xtrain)∑weight(xtrain)(xtest∈Xtest,xtrain∈Xtest,Class(xtrain)=Ci,1≤i≤n).

*Step 7*.  Classify each test sample to the class with highest similarity. Consider

(23) $$\begin{array}{c} \text{Class}\left(x_{\text{test}}\right)=\arg\max\left(\text{Similarity Class}\left(x_{\text{test}},C_{i}\right)\right). \end{array}$$

### 3.3. Node Influence Based Method 2 (NIM2)

Similarity Matrix is used twice in seven main steps of NIM1. The first is located in Step 4, in order to obtain the adjacency matrix. The second is in Step 6, in order to calculate the similarity between every test sample and each class. We believe in two steps used in different similarity matrix, resulting in node influence based method 2 (NIM2). Only two main steps of NIM2 are different from NIM1, as shown below.

*Step 3.* Set the parameter *δ*_1_; calculate the similarity between every two samples to construct the similarity matrix. Consider

(24) Similarity(x1,x2)=exp⁡(−Dist(x1,x2)2δ12) (x1,x2∈{Xtrain,Xtest}).

*Step 6*. Set the parameter *δ*_2_; calculate the similarity between every two samples and then obtain the similarity between every test sample and each class. Consider

(25) Similarity  2(x1,x2)=exp⁡(−Dist(x1,x2)2δ22) (x1,x2∈{Xtrain,Xtest}),Similarity Class(xtest,Ci)=∑Similarity  2(xtest,xtrain)·weight(xtrain)∑weight(xtrain)(xtest∈Xtest,xtrain∈Xtest,Class(xtrain)=Ci,1≤i≤n).

## 4. Experimental Results and Analysis

### 4.1. Benchmark Data Sets

We use 6 data sets to validate NIM1 and NIM2. Below are six publicly available gene expression data from DNA microarray that are widely used by researchers for cancer classification experiments. All the data sets are used to predict various kinds of cancers by measuring gene sequences and are outlined in Table 1.

The first data set is breast cancer [4]. The training data contains 78 patient samples, 34 of which are from patients who had developed distance metastases within 5 years. The remaining 44 samples are from patients who remained healthy from the disease after their initial diagnosis for an interval of at least 5 years.

The second data set is central nervous system [5]. Survivors are patients who are alive after treatment while the failures are those who succumbed to their disease. The data set contains 60 patient samples; 21 are survivors and 39 are failures. There are 7129 genes in the dataset.

The third data set is colon tumor [11]. It contains 62 samples gathered from colon-cancer patients. Among them, 40 tumor biopsies are from tumors and 22 normal biopsies are from healthy parts of the colons of the same patients. Two thousand out of around 6500 genes were selected founded on the confidence in the measured expression levels.

The fourth data set is prostate cancer [10]. The training set contains 52 prostate tumor samples and 50 nontumor prostate samples with around 12600 genes.

The fifth data set is acute lymphoblastic leukemia [12]. The data have been divided into six diagnostic groups and one that contains diagnostic samples that did not fit into any one of the above groups.

The sixth data set is lung cancer [6]. It is about the classification between malignant pleural mesothelioma (MPM) and adenocarcinoma (ADCA) of the lung. There are 181 tissue samples (31 MPM and 150 ADCA). The training set contains 32 of them, 16 MPM and 16 ADCA. The remaining 149 samples are used for testing. Each sample is characterized by 12533 genes.

If the dataset has not been divided into training set and testing set, we adopt leave-one-out cross validation (LOOCV) to validate NIM1 and NIM2. LOOCV involves using a single observation from the original sample as the validation data and the remaining observations as the training data. This is repeated such that each observation in the sample is used once as the validation data. This is the same as a K-fold cross-validation with K being equal to the number of observations in the original sampling.

### 4.2. Results

**Figure 7:**
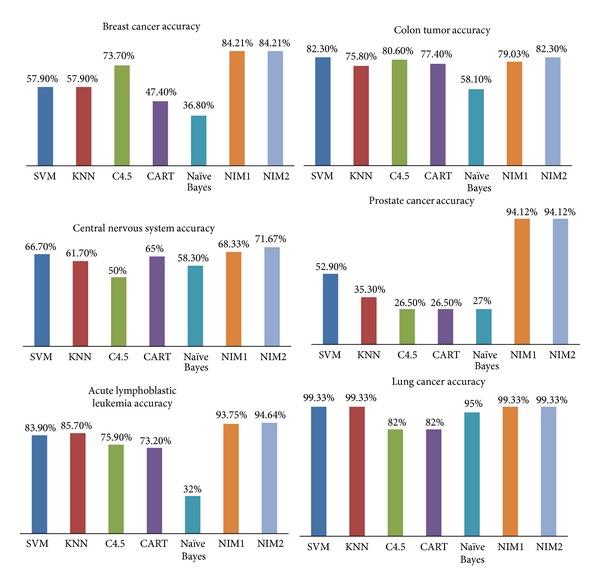
Experimental results for cancer gene datasets.

Most proposed cancer classification methods are from the statistical and machine learning area, ranging from the old nearest neighbor analysis to the new support vector machines. There is no single classifier that is superior over the rest. Some of the methods only work well on binary-class problems and are not extensible to multiclass problems, while others are more general and flexible. The methods we choose for comparing are all top 10 algorithms in data mining, mentioned in [13]. They are support vector machine (SVM) [14], *k*-nearest neighbor (KNN) [15], C4.5 [16], naive Bayes [17], and CART [18]. And we use the popular noncommercial open platform Weka (Waikato Environment for Knowledge Analysis) [19] for the implementation of the algorithms above. Experimental results on these six data sets using SVM, KNN, C4.5, Naive Bayes, NIM1, and NIM2 are presented in Figure 7.

Due to high dimension, small sample size, and nonbalanced distribution, traditional classification algorithms do not obtain high accuracy in these data sets. From Figure 6, we can see clearly that NIM1 obtain the highest accuracy in 5 of 6 data sets, and especially 94.12% in prostate cancer, compared to poor performance of other algorithms. And in colon tumor in which NIM1 does not get the highest accuracy, the performance of NIM1 differs very little with the highest one.

NIM2 is an improved version of NIM1 and has one more parameter. NIM1 can be viewed as a special case of NIM2 when *δ*_1_ = *δ*_2_. So the results of NIM2 are at least as good as NIM1. From Figure 7, we can see clearly that NIM2 obtain the highest accuracy in all 6 data sets. Thus, NIM1 and NIM2 are more efficient and robust than traditional classification algorithms in these cancer gene data sets.

### 4.3. Parameters Discussion

The traditional classification methods usually tend to have many parameters need to be set before application. And the parameters are closely related to the performance. However, there is little information on how to set parameters, usually based on experience. So we try to propose an algorithm with as few parameters as possible. NIM1 has only one parameter *δ*, and NIM2 has only two parameters *δ*_1_ and *δ*_2_.

**Figure 8:**
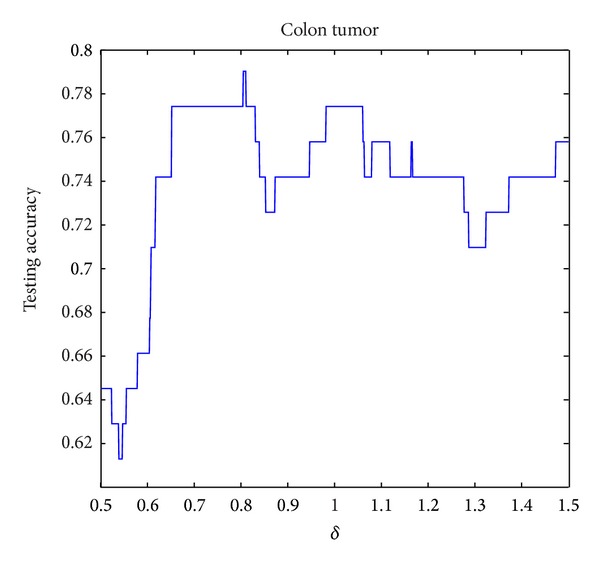
NIM1 results in colon tumor with the variation of *δ*.

**Figure 9:**
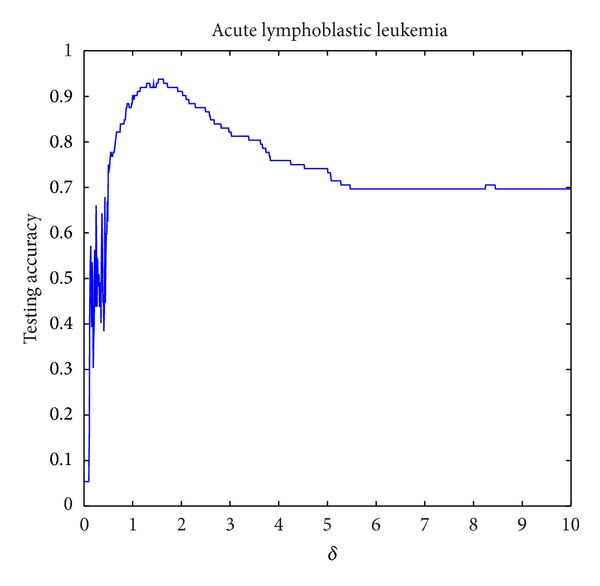
IM1 results in ALL with the variation of *δ*.

**Figure 10:**
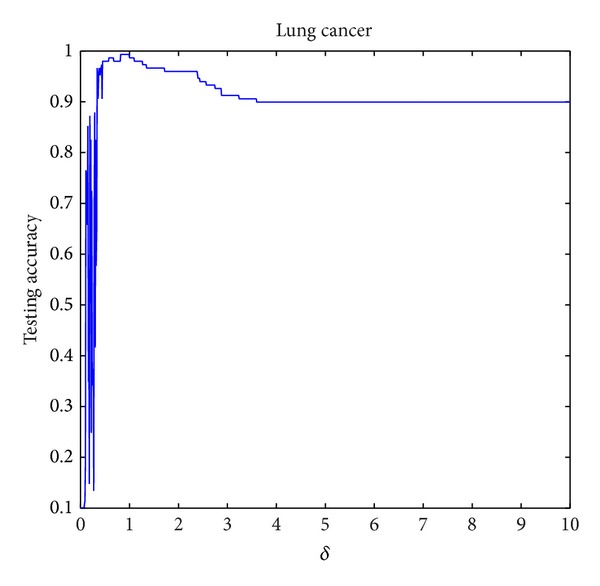
NIM1 results in lung cancer with the variation of *δ*.

**Figure 11:**
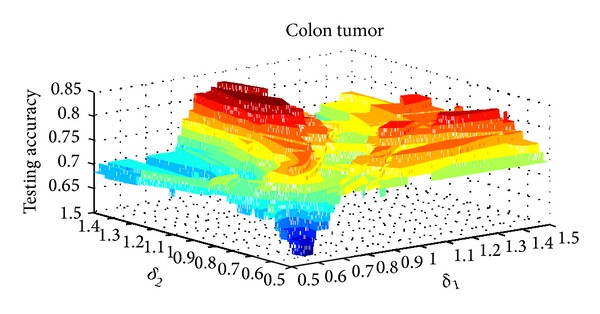
NIM2 results in colon tumor with the variation of *δ*_1_, *δ*_2_.

**Figure 12:**
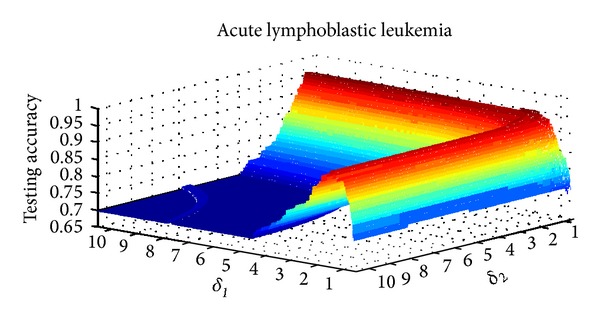
NIM2 results in ALL with the variation of *δ*_1_, *δ*_2_.

**Figure 13:**
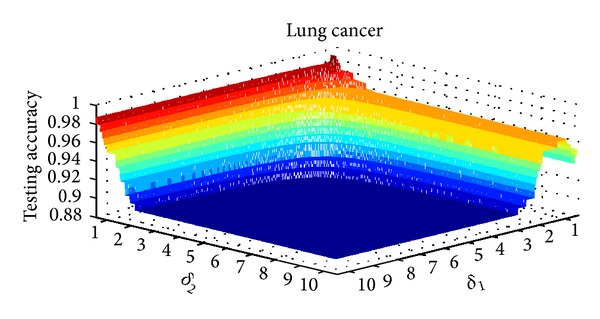
NIM2 results in lung cancer with the variation of *δ*_1_, *δ*_2_.

The parameter setting for *δ* in NIM1 is shown in Table 2, and parameters setting for *δ*_1_ and *δ*_2_ in NIM2 is shown in Table 3. Three data sets are selected for parameter variation experiments; they are colon tumor, acute lymphoblastic leukemia, and lung cancer. Figures 8, 9, and 10 show the results of NIM1 with the variation of *δ* in the 3 data sets. Figures 11, 12, and 13 show the results of NIM2 with the variation of *δ*_1_, *δ*_2_ in the 3 data sets. From the experimental results shown in Figures 11, 12, and 13, we can see clearly that both of the *δ*_1_ and *δ*_2_ play an important role in the performance of NIM2.

## 5. Conclusion

Graph is a powerful representation formalism that has been widely employed in machine learning and data mining. In order to gain deep insight into the cancer classification problem, we analyze the problem from graph-based view. Let *m* indicate the number of genes measured. Every cancer sample can be viewed as a point in *m*-dimensional space. And the set of cancer samples can be viewed as a graph (or network) in *m*-dimensional space.

In the method NIM1, after selecting the appropriate distance metric, the graph (or network) of all samples is created by computing the similarity matrix. Then the node influence of training samples is calculated. Treat node influence as weight; the similarity between every test sample and each class is obtained. At last, every test sample is classified according to its similarity between each class.

Furthermore, we also propose NIM2, which is an improved version of NIM1. NIM1 can be viewed as a special case of NIM2 when *δ*_1_ = *δ*_2_. Both NIM1 and NIM2 can deal with binary and multiclass cancer classification. NIM2 is more time consuming than NIM1 but owns a higher accuracy.

Due to high dimension, small sample size, and nonbalanced distribution, SVM, KNN, C4.5, Naive Bayes, and CART do not obtain high accuracy in these cancer gene data sets. From the experimental results in the 6 cancer gene data sets, it can be seen that NIM1 and NIM2 are more efficient than these traditional algorithms. At the end, we also discuss the parameters in both NIM1 and NIM2. The parameters play an important role in the performance of NIM1 and NIM2.

## Figures and Tables

**Table 1:** Description for cancer gene data sets.

Dataset	Number of samples	Number of genes	Number of classes	Test method
Breast cancer	97	24481	2	78train-19test
Central nervous system	60	7129	2	LOOCV
Colon tumor	62	2000	2	LOOCV
Prostate cancer	136	12600	2	102train-34test
Acute lymphoblastic leukemia	327	12558	7	215train-112test
Lung cancer	181	12533	2	32train-149test

**Table 2:** Parameter setting for δ in NIM1.

Dataset	Minimum of δ	Maximum of δ	Change interval	Number of experiments
Colon tumor	0.501	1.5	0.001	1000
Acute lymphoblastic leukemia	0.01	10	0.01	1000
Lung cancer	0.01	10	0.01	1000

**Table 3:** Parameters setting for *δ*_1_, *δ*_2_ in NIM2.

Dataset	Minimum of δ_1_, δ_2_	Maximum of δ_1_, δ_2_	Change interval of δ_1_, δ_2_	Number of experiments
Colon tumor	0.501, 0.501	1.5, 1.5	0.001, 0.001	1000000
Acute lymphoblastic leukemia	0.501, 0.501	10.5, 10.5	0.01, 0.01	1000000
Lung cancer	0.501, 0.501	10.5, 10.5	0.01, 0.01	1000000
